# Symmetry of ictal slow waves may predict the outcomes of corpus callosotomy for epileptic spasms

**DOI:** 10.1038/s41598-019-56303-3

**Published:** 2019-12-24

**Authors:** Sotaro Kanai, Masayoshi Oguri, Tohru Okanishi, Shinji Itamura, Shimpei Baba, Mitsuyo Nishimura, Yoichiro Homma, Yoshihiro Maegaki, Hideo Enoki, Ayataka Fujimoto

**Affiliations:** 1 Department of Child Neurology, Seirei-Hamamatsu General Hospital, 2-12-12 Sumiyoshi, Hamamatsu, 430-8558 Japan; 2 Division of Child Neurology, Institute of Neurological Sciences, Faculty of Medicine, Tottori University, 86 Nishimachi, Yonago, 683-8503 Japan; 3 Division of Pathobiological Science and Technology, School of Health Sciences, Faculty of Medicine, Tottori University, 86 Nishimachi, Yonago, 683-8503 Japan; 4 Laboratory of Neurophysiology, Seirei-Hamamatsu General Hospital, 2-12-12 Sumiyoshi, Hamamatsu, 430-8558 Japan; 5 Department of General Internal Medicine, Seirei-Hamamatsu General Hospital, 2-12-12 Sumiyoshi, Hamamatsu, 430-8558 Japan; 6 Comprehensive Epilepsy Center, Seirei-Hamamatsu General Hospital, 2-12-12 Sumiyoshi, Hamamatsu, 430-8558 Japan

**Keywords:** Epilepsy, Epilepsy

## Abstract

We aimed to analyse the ictal electrographic changes on scalp electroencephalography (EEG), focusing on high-voltage slow waves (HVSs) in children with epileptic spasms (ES) and tonic spasms (TS) and then identified factors associated with corpus callosotomy (CC) outcomes. We enrolled 17 patients with ES/TS who underwent CC before 20 years of age. Post-CC Engel’s classification was as follows: I in 7 patients, II in 2, III in 4, and IV in 4. Welch’s t-test was used to analyse the correlation between ictal HVSs and CC outcomes based on the following three symmetrical indices: (1) negative peak delay: interhemispheric delay between negative peaks; (2) amplitude ratio: interhemispheric ratio of amplitude values for the highest positive peaks; and (3) duration ratio: interhemispheric ratio of slow wave duration. Ages at CC ranged from 17–237 months. Four to 15 ictal EEGs were analysed for each patient. The negative peak delay, amplitude ratio and duration ratio ranged from 0–530 ms, 1.00–7.40 and 1.00–2.74, respectively. The negative peak delay, amplitude ratio and duration ratio were significantly higher in the seizure residual group (*p* = 0.017, <0.001, <0.001, respectively). Symmetry of ictal HVSs may predict favourable outcomes following CC for ES/TS.

## Introduction

Epileptic spasms (ES) are epileptic seizures characterised by brief muscle contractions that typically involve the axial muscles and proximal limb segments^1^. The prolonged muscle contractions following typical ES are defined as tonic spasms (TS)^2^. Previous studies have identified three contiguous patterns of ictal electroencephalography (EEG) findings during spasms: (1) fast waves or high-frequency oscillations (HFOs) preceding the ES, (2) high-voltage slow waves (HVSs) and (3) desynchronization^3–5^. Among these, HVSs coincide with the phasic motor component of the ES in almost 100% of cases^1,5^. The pathological mechanisms giving rise to ES remain largely unknown. Previous research has indicated that both the cortex and subcortical structures including the brainstem, thalamus and basal ganglia play a role in the emergence of ES^6,7^. Researchers have hypothesised that ES are derived from cortical-subcortical interactions wherein the cortex triggers the activation of subcortical structures, thereby leading to the generation of ES^3,7^.

Corpus callosotomy (CC) is a valuable palliative surgical option for patients with diffuse or multifocal epileptic discharge resulting in generalised seizures^8,9^. The pathophysiologic basis of CC is the hypothesis that the corpus callosum is the most important pathway for the spread of epileptic activity between the two hemispheres of the brain^10^. Some reports have indicated that CC exerts beneficial effects in patients with ES^11–13^. In these studies, ES were eliminated after CC in 42 of 87 patients. Ono *et al*. proposed that interhemispheric recruitment of the epileptogenic state via the corpus callosum accounts for bilateral synchrony^14^, and that disconnection of the transcallosal volleys may lead to seizure elimination in such cases.

Buchmann *et al*. demonstrated that activity in the corpus callosum is significantly correlated with maximal slow wave activity, suggesting that callosal connections play a role in the synchronization of slow cortical activity in the bilateral hemispheres^15^. Based on this finding, we hypothesised that greater corpus callosum involvement would be associated with increased synchrony of HVSs and disconnection of the corpus callosum would resolve ES/TS especially in patients exhibiting symmetrical HVSs. In the present study, we retrospectively analysed the ictal EEGs of patients with ES/TS, focusing on symmetrical indices in patients exhibiting HVSs, in order to identify prognostic factors for CC.

## Results

### Patient characteristics

We recruited 17 patients (15 male, 2 female) who fulfilled the inclusion criteria. Their clinical profiles are described in Table 1. The mean age at epilepsy onset was 23 months (range: 1–166 months) and the mean age at CC was 81.4 months (range: 17–237 months). Three patients underwent anterior four-fifths CC, whereas the remaining 14 underwent total CC. The mean follow-up period after CC was 22.1 months (range: 8–72 months). Engel’s classification was I in seven patients, II in two patients, III in four patients and IV in four patients.

**Table 1:** Clinical information of patients with favourable (free from ES/TS) and unfavourable (others) outcomes.

	ES/TS free N = 7	ES/TS residual N = 10	*p*-value
Sex (boys: girls)	6: 1	9: 1	n.s.
Types of epilepsy syndrome			n.s.
West syndrome	2	8	
Symptomatic generalised epilepsy	5	2	
Aetiology			n.s.
Structural abnormality	5	6	
Genetic/chromosomal syndrome	2	1	
Unknown	0	3	
Age at epilepsy onset [months, range (mean)]	4–166 (49)	1–13 (5)	n.s.
Total number of AEDs before CC [range (mean)]	4–8 (6.6)	6–10 (7.3)	n.s.
Frequency of ES/TS			n.s.
1–20/day	5	5	
>20/day	2	5	
Age at CC [months, range (mean)]	45–237 (125)	17–106 (51)	0.042
Procedure of CC			n.s.
Total callosotomy	6	8	
Anterior 4/5 callostomy	1	2	
Outcomes of Engel’s classification			NA
I	7	—	
II	—	2	
III	—	4	
IV	—	4	
Follow-up periods [months, range (mean)]	8–36 (17)	10–72 (26)	n.s.

ES, epileptic spasms; TS, tonic spasms; AEDs: antiepileptic drugs; CC, corpus callosotomy; n.s., not significant; NA, not applicable. We used Fisher’s exact probability test, Welch t-test and chi-square test, appropriately. Tuberous sclerosis complex was classified to structural abnormality in aetiology.

Nine patients were diagnosed with West syndrome, six with symptomatic generalised epilepsy (SGE) and one with symptomatic localization-related epilepsy. All but patients 4, 8 and 17, who developed only ES/TS, experienced more than two types of seizures, including tonic seizures, complex partial seizures, atonic seizures and myoclonic seizures. The mean frequency of ES/TS was 40.1 times per day (range: 5–200).

Symptomatic aetiologies were identified in 16 patients. Patient 3 had both TSC and acute encephalopathy, whereas patient 7 had both *MECP2* duplication syndrome and acute encephalopathy. Three patients had no neurologic history prior to the onset of epilepsy.

### Ictal HVSs distribution

EEG data were reviewed for 201 ES/TS cases. In total, 46 ES/TS cases were excluded due to non-negligible artifacts or misplacement of EEG electrodes, following which we analysed the remaining 155 ES/TS cases. Four to fifteen ictal EEGs were reviewed for each patient (mean: 9.1). Main ictal slow waves tended to present in the occipital area for patients 1, 3, 4, 7, 12 and 13; in the temporal area for patients 2, 5, 6, 8, 9 and 16; in the frontal area for patients 11 and 15; and in the central-parietal area for patients 10, 14 and 17. Among all 155 ictal EEGs, seven were analysed between both front polar electrodes, whereas nine were frontal, 13 were central, 14 were parietal, 50 were occipital, 24 were anterior-temporal, 14 were mid-temporal and 24 were posterior-temporal.

### Symmetrical indices and other factors

The negative peak delay ranged from 0–530 ms (mean: 77.0 ms), the amplitude ratios ranged from 1.00–7.40 (mean: 1.61) and the duration ratios ranged from 1.00–2.74 (mean: 1.24). The entire data of EEG and electromyography (EMG) analyses can be found in Supplementary Table S1. Fast wave bursts preceding the ictal slow wave occurred only in patients 5, 7, 10 and 15, although they were not consistent even in these patients. The EMG latency ranged from 0–540 ms (mean: 115.8 ms), whereas the EMG delay ranged from 0–326 ms (mean: 56.1 ms).

### Statistical results

Data on clinical information were also collected from the patients. The analyses revealed that the age at CC was higher (*p = *0.042) in the patients free from ES/TS than in the patients with residual ES/TS.

**Figure 1:**
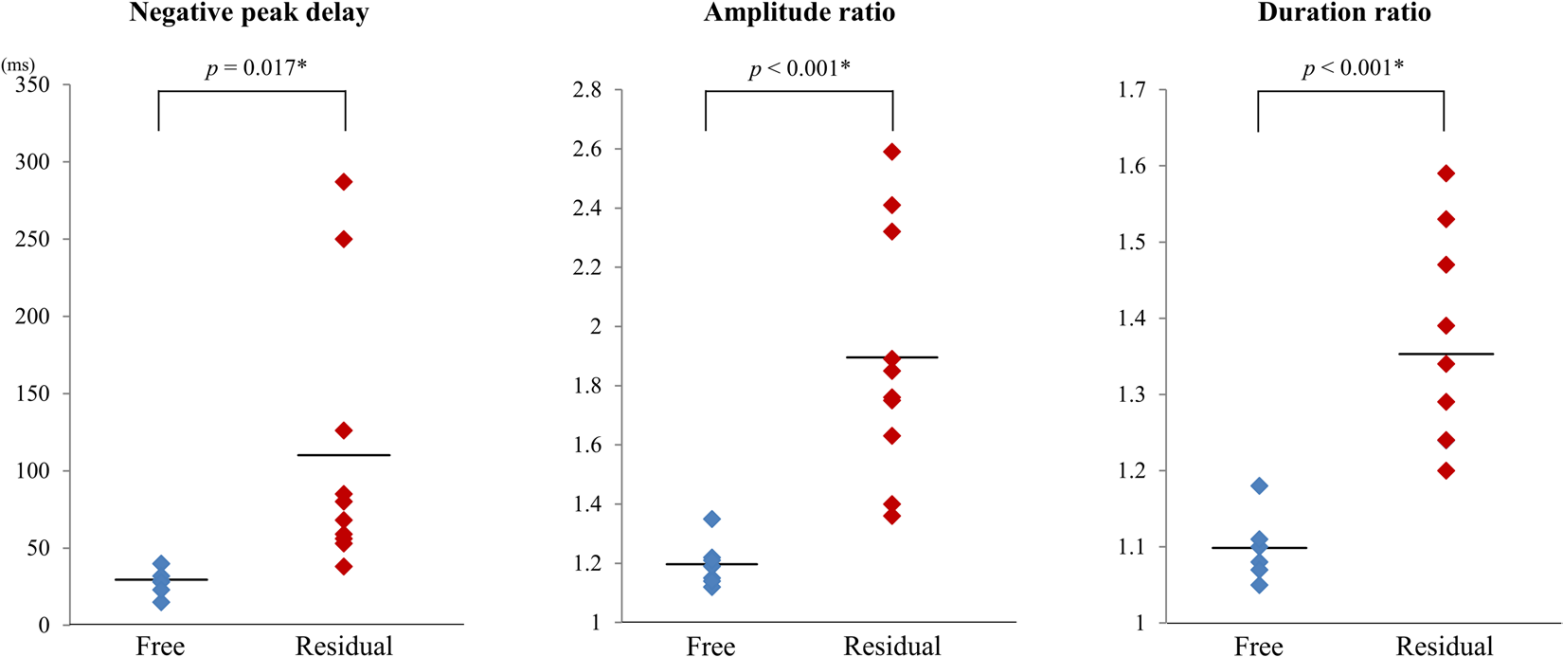
Plots of the symmetrical indices in each outcome group. All three indices of negative peak delay (*p* = 0.017), amplitude ratio (*p* < 0.001) and duration ratio (*p* < 0.001) were significantly higher in the ES/TS residual group than in the ES/TS free group (Welch’s t-test).

For the symmetrical indices, all three indices of negative peak delay (*p* = 0.017), amplitude ratio (*p* < 0.001) and duration ratio (*p* < 0.001) were significantly higher in the ES/TS residual group than in the ES/TS free group (Fig. 1).

When differentiating between ES/TS free group and ES/TS residual group, the receiver operating characteristic (ROC) curves for the negative peak delay, amplitude ratio, duration ratio and age at CC had an area under the curve (AUC) of 0.97, 1.00, 1.00 and 0.84, respectively (Table 2). The optimal operating point for each index at the maximum value of Youden’s index (YI) was calculated from the ROC analysis and the corresponding sensitivity and specificity were determined. The best prognostic indices in the ES/TS free group were the amplitude ratio and the duration ratio. The sensitivity and specificity for the ES/TS free outcome group were 1.00 and 1.00, respectively.

**Table 2:** Cutoff values of symmetrical indices and age at corpus callosotomy for favourable outcomes.

	AUC	Cutoff value	Sensitivity for ES/TS free	Specificity for ES/TS free
Negative peak delay	0.97	40.00	1.00	0.90
Amplitude ratio	1.00	1.35	1.00	1.00
Duration ratio	1.00	1.18	1.00	1.00
Age at CC	0.84	69.00	0.71	0.80

ROC, receiver operating characteristic; AUC, area under the curve; ES, epileptic spasms; TS, tonic spasms; CC, corpus callosotomy.

According to the power analysis, the necessary sample size was 10 with a power of 0.989. The sample size in this study was above 10 and hence was thought to be sufficient for the investigation.

## Discussion

In this retrospective study, we investigated the correlations between the symmetry indices of ictal HVSs and CC outcomes in patients with ES/TS. The negative peak delay, amplitude ratio and duration ratio among the bilateral hemispheres were significantly higher in the patients with residual ES/TS than in those with ES/TS free outcome. Our findings revealed that asymmetry of ictal HVSs in ES/TS is correlated with insufficient efficacy of CC for seizures.

Previous studies have identified the characteristics of ictal EEG during ES, which consist of three contiguous components: fast waves or HFOs preceding the ES, HVSs and desynchronization of electrical activity^3–5^. Some authors have focused on the association between ES genesis and fast waves or HFOs, revealing that the distribution of HFOs is correlated with epileptogenic brain lesions^16–20^. In contrast, few studies have investigated ictal HVS, focusing primarily on their morphology and distribution^21,22^.

Previous studies have demonstrated that changes in HVSs represent the most essential aspects of EEG alteration associated with ES and that such waves coincide with the phasic motor component in almost 100% of cases^1,5^. Some researchers have hypothesised that HVSs are generated in the subcortical structures^1^, while others have suggested that they are generated in the cortex^21,23^. Kobayashi *et al*. proposed that neocortical activation of HVSs occurs according to their complex and localised distribution^21^. Given the intimate relationship between ictal HVSs and ES, we focused on their characteristics and sites of origin in order to elucidate the pathophysiology of ES. Thus, we also investigated the association between HVSs and CC outcomes in patients with ES/TS.

Some previous studies have demonstrated the efficacy of CC for alleviating ES^11–13^. Pinard *et al*. reported that total CC was associated with greater seizure reduction than was anterior two-thirds CC in patients with West syndrome^11^. Conversely, other authors have reported that CC may be ineffective for reducing ES^22,24^, suggesting that some forms of ES are less affected by the corpus callosum. Furthermore, in one case report, a patient with West syndrome whose ES were initially bilateral and synchronous developed bilaterally independent ES originating in either hemisphere after total CC^25^. ES are speculated to represent a heterogeneous group of seizures that derive from a peculiar age-related cortical-subcortical interaction: Vigevano *et al*. expressed that ES appear to represent a sort of “final common manifestation”^3^.

Previous studies have demonstrated prognostic factors of resection surgery for neocortical epilepsy and temporal lobe epilepsy^26–28^. These studies described that intracranial EEG findings could help determine resection planning and predict outcomes. In contrast, limited studies have investigated prognostic factors of CC. In patients with SGE and frontal lobe epilepsy who experience tonic seizures, atypical absences, drop attacks, atonic seizures, generalised tonic-clonic seizures and myoclonic seizures, the degree of the morphologic similarity of the bilateral spike-waves in presurgical scalp EEG has been reported to correlate with excellent outcomes following anterior CC^29^. Although the efficacy of CC for ES/TS has been clarified in recent years^11–13^, no studies have investigated prognostic factors that can be used to predict outcomes. Thus, our findings are the first to demonstrate that presurgical electrophysiological parameters can be used as prognostic indicators for CC in patients with ES/TS. Additionally, another advantage of our study is the low invasiveness of scalp EEG. Since CC occasionally causes serious complications such as haemorrhage, appropriate selection of patients using presurgical non-invasive data is essential.

Avanzini *et al*. reported that interhemispheric coherence of fast activity was low prior to ES and that such activity increases following ES, suggestive of signal transfer through the corpus callosum during the sequential ES state^6^. Ono *et al*. proposed greater involvement of the corpus callosum, suggesting that an interhemispheric recruitment via the corpus callosum precedes cortical spike discharges, maintaining cortical epileptogenic susceptibility in both hemispheres^14^.

However, the functional roles of the corpus callosum in generating ictal HVSs associated with ES remain to be identified. Previous report has indicated that the corpus callosum is significantly correlated with maximal slow wave activity during non-rapid eye movement (NREM) sleep, indicating that callosal connections may play a role in the synchronization of slow cortical activity in the bilateral hemispheres^15^. The contribution of the corpus callosum for coherent activity during NREM sleep is further illustrated by the reduced interhemispheric coherence observed in children with congenital agenesis of the corpus callosum^30^. Among three symmetrical indices in the present study, the negative peak delay may highly reflect the extent of interhemispheric synchrony, as it may indicate the time difference of cortical-subcortical activation between both hemispheres. The low amplitude ratio and the duration ratio may reflect the approximation of epileptic excitations among bilateral hemispheres via the transcallosal volleys. Further studies involving larger numbers of cases are required to determine the index that is the best prognostic factor for CC outcomes.

Given that ES are completely eliminated by callosal disconnection in some patients, the corpus callosum may play a role in the generation of symmetrical ictal HVSs, which are regarded to be the most strictly associated with motor phenomena. The functional roles of the corpus callosum that intensify epileptogenesis may be more prominent in patients who respond favourably to CC. Interhemispheric recruitment via the corpus callosum may occur in patients with ES/TS who present with symmetrical HVSs due to bihemispheric synchrony, which may be eliminated via CC.

The present study has some limitations of note. Since the number of patients were not enough to perform multivariate analyses in this study, we could not evaluate the effect of age at CC for the symmetrical indices or identify the best prognostic factor among the indices. We visually selected the main ictal HVSs using AP-bipolar and average references. For more objective analysis, further studies should utilise additional approaches, such as mechanical frequency analysis.

Outcomes following CC for ES/TS are diverse among patients. We hypothesised that the patients presenting with favourable outcomes following CC may exhibit intense involvement of the corpus callosum in the generation of ES/TS. Thus, we focused on the synchrony of the ictal HVSs. Our findings indicated that the symmetry of the ictal HVSs for ES/TS was correlated with favourable CC outcomes. These findings indicated that the corpus callosum may be involved in the generation of symmetrical HVSs associated with ES/TS. Further studies regarding the pathophysiology of ES are required in order to develop appropriate treatment strategies.

## Methods

### Patients

Clinical data and video EEG recordings were retrospectively collected from each patient’s medical records. Inclusion criteria were as follows: (1) CC performed in patients at age of < 20 years between 2008 and 2017 at Seirei-Hamamatsu General Hospital, (2) main seizure type of ES and/or TS, (3) availability of ictal EEG recordings prior to CC, (4) ictal EEG containing polyphasic HVS and (5) follow-up period after CC greater than 6 months. Patients with inappropriate EEG recordings (e.g., misplacement of EEG electrodes) or serious surgical complications were excluded from the analysis.

The present study was approved by the institutional review boards of Seirei-Hamamatsu General Hospital and Tottori University. In accordance with approved guidelines, patient information including age, seizure type, aetiology, EEG data and postsurgical seizure outcome was anonymised prior to our analysis. Informed consent was obtained from each subject’s guardian.

### Clinical profiles

For each patient, we reviewed clinical data including sex, age at epilepsy onset, number of antiepileptic drugs (AEDs) prescribed prior to CC, frequency of ES/TS, classification of epilepsy syndrome, aetiology, age at CC, CC procedure and follow-up period. We defined the patients with ES/TS and developmental delay as having SGE or symptomatic localization-related epilepsy in accordance with seizure types and the distribution of EEG discharge, based on the 1989 International Classification of Epileptic Syndromes^31^.

Seizure outcomes following CC were evaluated for the remission of ES/TS based on Engel’s classification at the last follow-up^32^.

### Scalp video-EEG recordings

Scalp video-EEG data were recorded using a NicoletOne or BMSI6000 system (Natus Medical Incorporated, WI) for patients 1–4, 6–9 and 11–16 and a Neurofax system (Nihon-Kohden, Japan) for patients 5, 10 and 17. EEG electrodes were placed in accordance with the international 10–20 scalp-electrode position. The sampling rate was set at 256 Hz (patients 3, 8, 12 and 16), 400 Hz (patients 1, 2, 4, 7, 11, 14 and 15), 500 Hz (patients 5, 10 and 17), 512 Hz (patients 6 and 13), or 1,024 Hz (patient 9). Low-cut and high-cut filters were set at 0.5 Hz and 70 Hz for NicoletOne or BMSI6000 and 1.6 Hz and 60 Hz for Neurofax. EMG electrodes were placed on both deltoid muscles.

### Ictal EEG analyses

**Figure 2:**
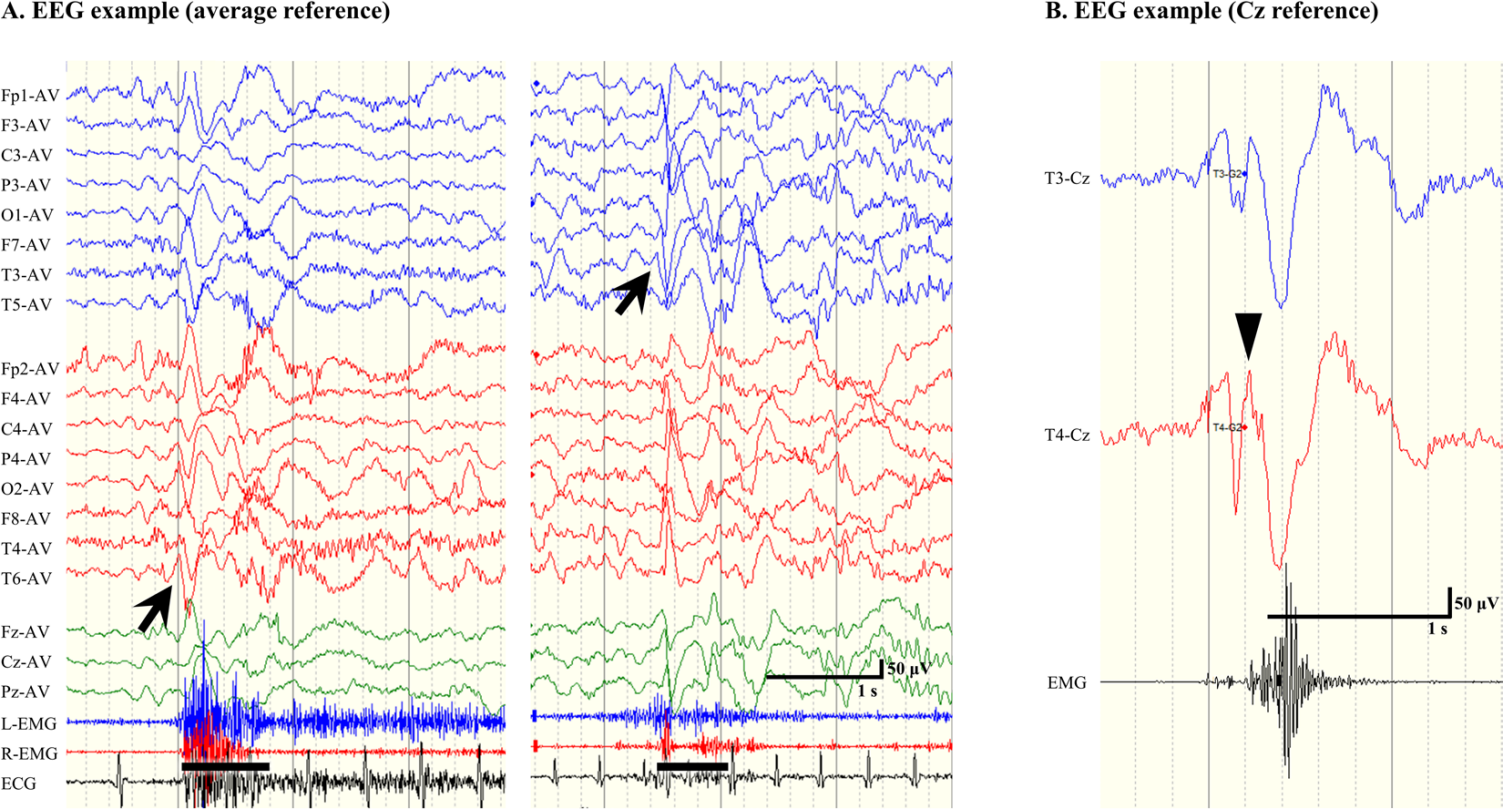
Representative examples of electroencephalography (EEG) findings for epileptic spasms (ES). (**A**) Ictal EEGs in an average reference montage. Symmetrical ictal high-voltage slow waves (HVSs; left) and asymmetrical ictal HVSs (right). The arrows indicate the main ictal HVSs. The bars represent the durations of visually confirmed motor phenomena. (**B**) Ictal EEG in Cz reference montage. The main ictal HVS emerges at T4 and is thus contrasted with T3. The wedge indicates the initial negative peak.

We retrospectively analysed the correlation between ictal polyphasic HVSs on scalp EEG and CC outcomes. First, we selected the main ictal HVS, which was defined as that occurring immediately prior to or simultaneously with ictal muscle contractions and presenting with the typical negative-positive-negative waveform^5^. Main ictal HVSs were analysed for each ES/TS based on the montages of AP-bipolar and average reference (Fig. 2A). Subsequently, we contrasted the main ictal HVS with the contralateral electrode using the Cz reference montage, in order to avoid excessive influence of a single hemisphere (Fig. 2B). We measured the time delay of initial negative peaks in each hemisphere, the amplitude of the highest positive peak of polyphasic slow waves and the duration of positive slow waves.

### Definitions of symmetrical indices and other factors

**Figure 3:**
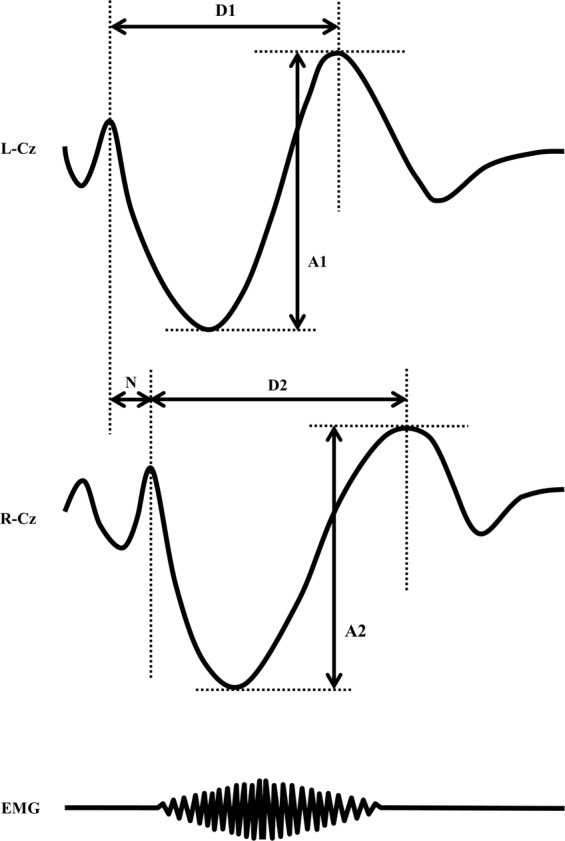
Schema of three symmetrical indices. N, negative peak delay (ms); A1(higher)/A2(lower), amplitude ratio; D2(longer)/D1(shorter), duration ratio.

In this study, we defined three symmetrical indices as follows (Fig. 3):

1. *Negative peak delay (ms)*: time delay of initial negative peaks among bilateral hemispheres [(preceding peak) – (following peak)]
2. *Amplitude ratio*: amplitude ratio of the highest positive peaks of polyphasic slow waves among bilateral hemispheres [(higher amplitude)/(lower amplitude)]
3. *Duration ratio*: duration ratio of positive slow waves among bilateral hemispheres [(longer duration)/(shorter duration)].

In addition, we measured EMG latency (latency from the initial negative peak to the onset of EMG burst) and EMG delay (time delay among bilateral EMGs). Each value was measured for individual seizures, following which average values were obtained for each patient.

### Statistical analyses

We divided the patients into two outcome groups of free from ES/TS (Engel’s I) or residual ES/TS (Engel’s II–IV). We statistically analysed the differences in clinical information using Fisher’s exact probability test, Welch’s t-test and chi-square test, appropriately.

We also statistically analysed the differences in three symmetrical indices of negative peak delay, amplitude ratio and duration ratio, using Welch’s t-test. Thereafter, we examined the prognostic values regarding covariates that were significantly associated with the outcomes, by analysis of ROC curves. The optimal operating point was determined via YI. The sensitivity and specificity of each operating point in the ROC curve and each index were conditionally calculated, followed by calculation of YI at each point (YI = sensitivity + specificity - 1).

We also examined whether the sample size of this study was enough. The power (z test with arc sin transformation) was calculated based on a hypothesis test of binomial probability. We set the alpha error as 0.05, beta error as 0.20, the power as 0.95 and effect size as 0.65, considering that CC is the last resort for pharmacologically intractable epilepsy with ES/TS.

The analyses were performed using JMP (version 12.0; SAS Institute Japan Inc., Tokyo) and R.3.6.1. The level of statistical significance was set at *p* < 0.05.

## Supplementary information

Supplementary information 

## Data Availability

The datasets generated and analysed during the current study are available from the corresponding author on reasonable request.
