# Supplementary material for: Symmetry of ictal slow waves may predict the outcomes of corpus callosotomy for epileptic spasms
Source: Sci Rep. 2019 Dec 24;9:19733. doi: 10.1038/s41598-019-56303-3 (PMC6930281; doi:10.1038/s41598-019-56303-3)
Supplement: Supplementary file 1 — Supplementary information [file 41598_2019_56303_MOESM1_ESM.pdf]

Symmetry of ictal slow waves may predict the outcomes of corpus callosotomy for epileptic spasms

Sotaro Kanai, Masayoshi Oguri, Tohru Okanishi, Shinji Itamura, Shimpei Baba,
Mitsuyo Nishimura, Yoichiro Homma, Yoshihiro Maegaki, Hideo Enoki, Ayataka Fujimoto

Supplementary Table S1. Results of EEG and EMG analyses

Patient	Number of ES/TS	Main distribution	Mean negative peak delay (ms)	Mean amplitude ratio	Mean duration ratio	Mean EMG latency (ms)	Mean EMG delay (ms)	Outcome of Engel's classification
1	10	Occ.	40	1.15	1.18	115	46.4	I
2	6	Temp.	40	1.12	1.1	63	77.2	I
3	7	Occ.	29	1.35	1.1	40	47.9	I
4	11	Occ.	15	1.22	1.08	121	26.5	I
5	10	Temp.	32	1.19	1.05	19	18.6	I
6	5	Temp.	28	1.21	1.11	134	13	I
7	15	Occ.	23	1.14	1.07	216	81.3	I
8	10	Temp.	68	1.89	1.59	104	44.9	II
9	9	Temp.	250	1.4	1.29	122	72.3	II
10	11	C-P	56	1.63	1.2	111	57.5	III
11	7	Front.	80	2.59	1.39	177	27.1	III
12	12	Occ.	126	1.75	1.24	145	142.3	III
13	4	Occ.	38	1.36	1.24	95	57	III
14	11	C-P	59	2.32	1.53	31	57.6	IV
15	4	Front.	85	1.76	1.24	93	68.8	IV
16	11	Temp.	287	1.85	1.47	215	58.7	IV
17	12	C-P	52.9	2.41	1.34	168	56	IV

EEG, electroencephalogram; EMG, electromyography; ES, epileptic spasms; TS, tonic spasms; Occ., occipital; Temp., temporal; C-P, central-parietal; Front., frontal.
